# Decoding Face Information in Time, Frequency and Space from Direct Intracranial Recordings of the Human Brain

**DOI:** 10.1371/journal.pone.0003892

**Published:** 2008-12-09

**Authors:** Naotsugu Tsuchiya, Hiroto Kawasaki, Hiroyuki Oya, Matthew A. Howard, Ralph Adolphs

**Affiliations:** 1 Division of Humanities and Social Sciences, Caltech, Pasadena, California, United States of America; 2 Department of Neurosurgery, University of Iowa, Iowa City, Iowa, United States of America; 3 Department of Neurology, University of Iowa, Iowa City, Iowa, United States of America; 4 Division of Biology, Caltech, Pasadena, California, United States of America; Victoria University of Wellington, New Zealand

## Abstract

Faces are processed by a neural system with distributed anatomical components, but the roles of these components remain unclear. A dominant theory of face perception postulates independent representations of invariant aspects of faces (e.g., identity) in ventral temporal cortex including the fusiform gyrus, and changeable aspects of faces (e.g., emotion) in lateral temporal cortex including the superior temporal sulcus. Here we recorded neuronal activity directly from the cortical surface in 9 neurosurgical subjects undergoing epilepsy monitoring while they viewed static and dynamic facial expressions. Applying novel decoding analyses to the power spectrogram of electrocorticograms (ECoG) from over 100 contacts in ventral and lateral temporal cortex, we found better representation of both invariant and changeable aspects of faces in ventral than lateral temporal cortex. Critical information for discriminating faces from geometric patterns was carried by power modulations between 50 to 150 Hz. For both static and dynamic face stimuli, we obtained a higher decoding performance in ventral than lateral temporal cortex. For discriminating fearful from happy expressions, critical information was carried by power modulation between 60–150 Hz and below 30 Hz, and again better decoded in ventral than lateral temporal cortex. Task-relevant attention improved decoding accuracy more than10% across a wide frequency range in ventral but not at all in lateral temporal cortex. Spatial searchlight decoding showed that decoding performance was highest around the middle fusiform gyrus. Finally, we found that the right hemisphere, in general, showed superior decoding to the left hemisphere. Taken together, our results challenge the dominant model for independent face representation of invariant and changeable aspects: information about both face attributes was better decoded from a single region in the middle fusiform gyrus.

## Introduction

Faces are processed by a relatively dedicated but anatomically distributed system. This proposition has received strong convergent support from intracranial recordings in humans [1]–[5], [6], [7], [8] as well as from a large number of imaging studies [9]–[12], scalp EEG [5], [13]–[17] and MEG studies [18], [19], in addition to lesion studies in humans [20], [21] and neurophysiological studies in monkeys [22]–[25]. While debates about the modularity of face processing continue [26], [27] there is consensus in the notion of a face-processing system that encompasses specific sectors of temporal visual cortex.

Distinct facial attributes, such as emotional expression, gender, and identity, are extracted through this face processing system in partly segregated functional streams [28]–[31]. In particular, it is thought that while static aspects of a face, such as its gender and identity, are encoded primarily in the ventral temporal regions, dynamic information, such as emotional expression, depends on the lateral and superior regions in the superior temporal sulcus and gyrus [9], [24], [25], [28]. This functional division of labor also meshes well with a dominant and influential model of face processing, which argues that faces need to be identified regardless of their expression, and that emotional expressions must often be recognized across different identities. Based in large part on this idea as well as behavioral data, the model proposes that identity and emotional expression information are processed by separate systems [32]. Recently, functional imaging data has buttressed this model, suggesting that invariant aspects of faces, including identity, are represented in the fusiform face area (FFA) [10], [33], in the ventral temporal cortex, while changeable aspects of faces, including emotional expressions, are represented in regions around the superior temporal sulcus (STS) [28]. However, a recent update to this model argues that there is early common processing of invariant and changeable facial attributes within the ventral temporal cortex, whose outputs are then conveyed to multiple cortical regions for further processing of distinct attributes [30], [34]. Given these alternative hypotheses, it is of special interest to contrast the information represented within the ventral temporal cortex with that represented in the lateral temporal cortex, and to examine the issue at a more precise resolution in time and frequency.

Just as information about faces is spatially distributed across cortical sites [9], [11], [12], [22], [28], faces are processed at various temporal scales. Event-related potentials (ERP) measured by scalp EEG and MEG have shown that the visual system classifies a stimulus category rapidly within around 100 msec based on the visual characteristics of the input [35], [36]. Faces, in particular, evoke activity in the fusiform gyri at around 170 msec, reflecting more detailed processing about various aspects of faces [1]–[3], [5], [8], [13]–[19]. Further face processing includes cognitive and emotional evaluation, linking conceptual knowledge signaled by the faces. Such later face processing would involve many subcortical structures such as amygdala, basal ganglia, hypothalamus, brain stem, as well as cortical areas such as orbitofrontal, somatosensory, and insular cortices [7], [31]. These prior findings leave open several important questions: Exactly what aspect of faces is encoded at early and at late latencies? Which regions of cortex participate in such encoding? And how does information flow from one region to another within the network?

The spatiotemporal complexity of face processing poses methodological difficulties in obtaining rich descriptions of how, and what point in time, different regions represent facial information. Moderately good spatial resolution and very wide field-of-view can be attained using fMRI, yet temporal resolution is limited to the timescale of seconds. Millisecond temporal resolution obtained using scalp EEG and MEG, on the other hand, is limited in terms of spatial resolution. Although direct single-unit recordings offer the best possible spatio-temporal resolution in principle, this technique suffers from an extremely narrow anatomical field-of-view together with very rare opportunities to obtain such recordings in humans [37]–[40]. Most importantly, none of these approaches provides a wide bandwidth such that different frequency components of processing could be adequately examined. Arguably the best combination of large anatomical field-of-view, good spatial resolution, excellent temporal resolution, and wide frequency bandwidth, is afforded by field potentials, which can be recorded in awake neurosurgical subjects [1]–[4], [6]–[8], [41]–[44].

**Figure 1:**
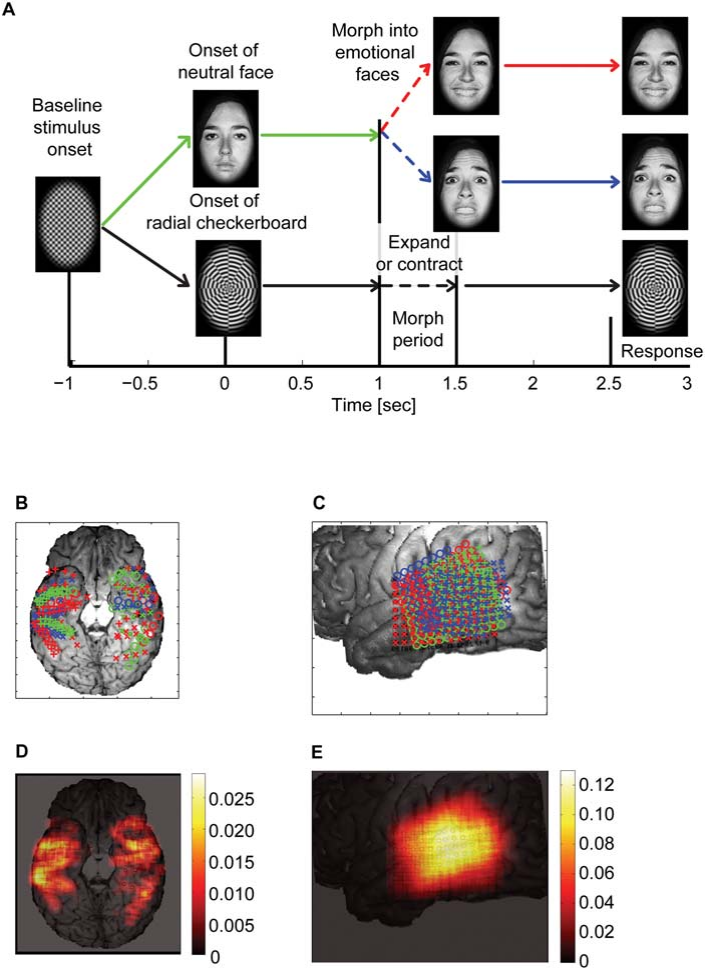
Experimental Paradigm. A We presented three kinds of morphing stimuli; 1) neutral to happy (80 trials), 2) neutral to fear (80 trials), 3) radial checkerboard (40 trials), the order of which was randomized within a session of 200 trials. A trial started with the baseline plaid pattern (−1<t<0). At *t* = 0, either a neutral face or a checkerboard was presented. At *t* = 1, the face started to morph into either fearful or happy, or the checkerboard expanded or contracted. After *t*>1.5, the stimuli remained frozen and a response was prompted at *t* = 2.5. Subjects performed either an emotion- or a gender- discrimination task with three alternatives (see main text) in a given session. 9 subjects were studied in 22 sessions. B and C Distribution of the electrodes in the ventral (B) and the lateral (C) cortex. The electrode placement for each subject is shown with a different symbol and color, superimposed on one representative subject's brain surface. D and E The electrode density map, representing the frequency of the electrode placement for all subjects. A faint outline of the brain is superimposed. See Methods for details of how the density map was computed.

To take a closer look at the face processing system in space, time and across frequency bands, we recorded intracranial multi-channel electrocorticograms (ECoG) from 9 subjects, who were performing a discrimination task on static and dynamic face stimuli (Figure 1). We analyzed the ECoG using a time-frequency decomposition. Time-frequency analyses allow much better preservation of information than the conventional event-related potential (ERP) of the raw ECoG (Figure S1 and S2), yet also introduce three large challenges. First, the data are high-dimensional (amplitude values defined at different time points at different frequencies in many channel locations). Second, the many concurrent recording channels require statistical corrections for multiple comparisons that severely limit statistical power. And third, inter-subject variation in electrode locations and the most responsive frequency ranges makes population-level inferences problematic, since it is unclear how to pool data across multiple subjects.

To address these problems, we applied a decoding approach to our time-frequency decomposed data. For example, combining the data across channels and frequencies within a subject, we can assess when information for emotion discrimination becomes available, effectively reducing the dimensions of the data and alleviating the multiple comparison problem. An optimal combination across channels blurs the exact locations of electrodes, solving the problem of inter-subject variation in sensitive electrode location and responsive frequencies.

Using this decoding approach, we were able to compare information processing in the ventral and the lateral temporal cortex. We found that higher decoding accuracy was obtained in the ventral than the lateral temporal cortex when we tried to discriminate faces from checkerboard patterns and fearful from happy expressions. Decoding time-frequency maps revealed critical frequency bands of 50–150 Hz for discrimination of faces from checkerboards not only when the stimuli were static, but also when the stimuli were morphing; in both cases, the decoding accuracy was better in the ventral than the lateral temporal cortex, consistent with a hypothesis arguing for early common face processing in the ventral temporal cortex. Further, emotion decoding was possible from 60–150 Hz and below 30 Hz, and better and faster in the ventral than the lateral temporal cortex.

## Results

### Behavioral results

We recorded electrophysiological responses from nine neurosurgical subjects by showing them either face or checkerboard stimuli and having them perform a three-alternative forced choice task on the stimuli by pressing a button (Figure 1a). In a given session of 200 trials, the three alternatives were either [1, happy; 2, other; 3, fear] or [1, woman; 2, other; 3, man]. We call a session with the former alternatives an “emotion discrimination session” and that with the latter a “gender discrimination session”. In any session, we randomly interleaved checkerboard stimuli in 40 trials. In these control trials, we instructed subjects to choose the alternative “other” to indicate the stimulus was not a face. For the rest of the 160 trials, we presented either a male or a female face (80 trials each) whose expression was neutral. After 1 second, we morphed the facial expression into either happy or fear over 0.5 seconds (40 trials each for male/happy, male/fear, female/happy, and female/fear). For those trials, we instructed subjects to choose the alternative that best described the stimulus. Behavioral responses were obtained in 19 of 22 sessions (3800 trials in total). The emotion and gender discrimination accuracy was 86.7±5.8% and 95.3±2.0% correct, respectively (mean+−s.e.m.), which were not significantly different (p>0.5).

### ERP and spectrogram analysis

We recorded field potentials from subdural electrodes that covered the ventral and the lateral temporal cortex (Fig. 1B–E). Before proceeding into our novel decoding approaches, we first describe an example of the results from more conventional ERP and spectrogram analyses. In Figure S1 and S2, we show the results from two responsive electrodes in the ventral temporal cortex (data from the first session for subject 153; for the locations of electrode 74 and 75, see Figure S3).

First, by averaging the raw field potentials in the time domain, we carried out an event-related potential (ERP) analysis (Figure S1A and S2A). At the abrupt onset of the static stimuli (t = 0 sec), field potentials were evoked in a stimulus-locked manner, resulting in larger positive or negative deflections to faces than to checkerboards at around 150–200 msec in many electrodes [1]–[4]. For the exemplar electrodes, t-scores from two-tailed t-tests (comparing face>checkerboard) exceeded t>6 (Figure S1B, red), t<−12 and t>14 (Figure S2B, red) around 200 msec from the onset of the static stimuli (uncorrected for multiple comparisons). In contrast, we rarely found such a clear ERP during the time interval of our dynamic emotion morph period (t = 1–1.5 sec). Most likely, because the stimuli were morphed smoothly over 500 msec, field potentials were not locked to the onset of the morph. As a result, we found very few ERPs (Figure S1A and S2A) that discriminated dynamic facial morphs from dynamic checkerboard movies (Figure S1B and S2B, red). In particular, we almost never found any strong ERP that discriminated fearful from happy expressions during the morph period (Figure S1B and S2B, blue).

Second, we estimated the event-related power spectrogram of the ECoG for each trial using a multi-taper spectral analysis [5], [6] and obtained the average of the spectrograms for each condition (Figure S1C–E and S2C–E). The multi-taper method involves the use of multiple data tapers (i.e., the prolate spheroidal Slepian functions) for spectral estimation, which stabilizes the estimate of the power spectrum over short segments of possibly non-stationary data, suited for an analysis of intracranial EEG. The estimated spectrograms showed well-known 1/f power distributions. In addition, for the exemplar electrode, the spectrograms for fearful and happy faces showed stronger evoked responses around 100 Hz just after the onset of the morph period (t = 1–2 sec), which was absent for checkerboards. T-scores from two-tailed t-tests revealed significantly larger responses to dynamic faces than moving checkerboards (uncorrected for multiple comparisons; 50–200 Hz, t = 1–2.5 sec, Figure S1F and S2F), and in particular, to fear morphs than to happy morphs (50–150 Hz, t = 1.2–2, Figure S1G). By limiting our frequency of interest to 50–150 Hz (high-gamma band) we found that the high-gamma response was enhanced by the appearance of static faces, but not by static full-contrast checkerboard patterns (t = 0.1–1 sec), and that it was disproportionately enhanced by fearful (Figure S1H, blue) rather than happy morphs (Figure S1H, red; recorded in channel 75 during the window 1.2<t<2 sec).

While the above analysis approach is commonly used in many EEG studies, it posed problems for our data. By applying t-tests at each time-frequency point, we faced massive multiple comparison problems. Even worse, we analyzed ∼100 electrodes and picked one of the best electrodes for Figures S1 and S2, further raising a concern for multiple comparisons. Strict correction, such as Bonferroni correction that assumes independent multiple hypothesis testing, would be unnecessarily strict because we often see strong correlation in signals across time, frequencies and neighboring electrodes. In the above approach, we defined the frequency of interest post-hoc; strictly speaking, our choice of frequency bands cannot be justified without prior independent studies. In practice, the best frequency bands were different from electrode to electrode, and from subject to subject. The best frequency bands also often depended on the testing condition. Thus, prior specification of a frequency band of interest could lead to poor statistical power for detecting any real positive effects. Or, if it is specified to maximize the effect in a particular study, it may over-fit the data and generalize poorly.

In order to address these problems, we applied multi-variate decoding analyses, which optimally linearly combined signals. Our decoding approaches objectively reduced the dimensionality of the signal, alleviating multiple comparison problems. A trained linear classifier learned correlations across time, frequency and electrodes in an appropriate way to optimize the decoding performance. By training the classifier with regularization [7] and validating the classifier against an untrained data set, we were able to retain high sensitivity with much less over-fitting. In the following, we describe our decoding approaches and the results obtained from the decoding analyses.

### Decoding analyses

As inputs to the linear classifier, we used the logarithm of the power estimated via the multi-taper method from each trial. We trained a regularized least-square classifier [7], [8] on randomly chosen 70% of the trials and tested its decoding performance on the remaining 30% of the trials for each session in each subject. We evaluated the decoding accuracy by submitting the classifier outputs into the receiver operating characteristic (ROC) analysis [9], rather than assigning a binary correct or incorrect label for each trial and computing % of correct classification. ROC analysis allowed us to utilize the information present in the magnitude of output from the classifier (i.e., an output close to 0 when inputs cannot be confidently classified as X or Y and an output far from 0 when inputs for a test trial is easily classified as X or Y). We submitted the graded classifier outputs for all test trials into the ROC analysis and computed the area under the ROC curve (Throughout the paper, we call the area under ROC curve A' for short).

**Figure 2:**
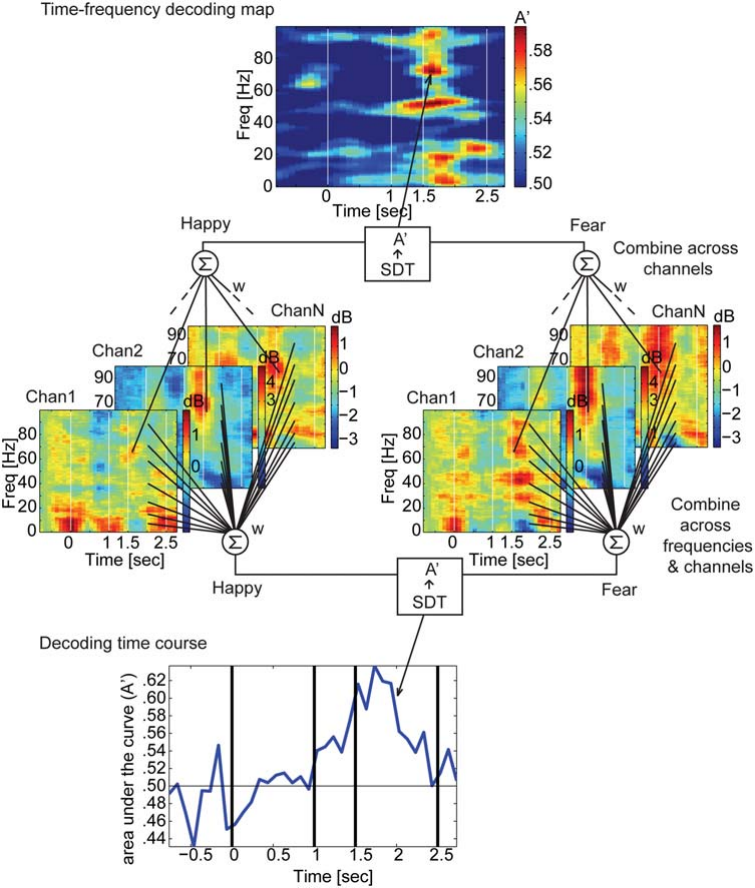
Decoding procedure. The different parts of the figure provide a schematic, using real data as an example, of how power spectrograms estimated in each electrode (the colored spectrograms in the middle) can be pooled to decode stimulus category (fear vs. happy in this example). (Middle) The average event-related spectrogram (colored graphs) was obtained for each electrode (“Chan1”…“ChanN”) under two different conditions (in this example, happy and fearful trials). Note that neighboring electrodes can show highly variable and complex responses at different frequencies (color-coded from −2 to +2 dB in channel 1, from −2 to +5 dB in channel 2, and from −3.5 to 1.5 dB in channel N). In the example depicted in the figure we show only 3 channels out of a typically much larger number, but the problem of visualization and statistical analysis is already apparent. (Top) Time-frequency decoding map for the ventral temporal cortex of one subject. Color code represents area under the ROC curve (A'). For example, the red pixel at 1.7 sec and 70 Hz (black arrow) means that when we combine the power at that time-frequency point from all the electrodes in the ventral temporal cortex with a linear weight (estimated from 70% of the training trials), the classifier can discriminate happy from fear with A' = .60 (or 60% correct classification with an arbitrary criterion) for the test trials. (Bottom) In order to characterize the latency of decoding, we combined the power across frequencies and electrodes. The peak decoding accuracy is A' = .64 for the bottom panel while it is A' = .60 in the top panel, showing an advantage in combining information across frequencies in addition to across electrodes. Decoding across frequencies also facilitates comparison across subjects because the peak of the sensitive frequency bands can vary across subjects but remain relatively constant in time across subjects.

Here we introduce three novel decoding approaches: 1) time-frequency decoding map, 2) time course of decoding, and 3) searchlight decoding. The time-frequency decoding map was obtained for each session in each subject by combining information across electrodes within a certain anatomical region at each time step at each frequency band with linear weights, taking into account the spatial correlation across electrodes. This map emphasizes the most informative time-frequency points, reducing the space dimension in an optimally linear manner. The time course of decoding for each session was obtained by combining information across electrodes (space) and frequencies, reducing the space and frequency dimensions in an optimally linear manner (Figure 2). The time course analysis provides the latency for decoding, an earliest estimate of the time when the information becomes available in a circumscribed anatomical region. Searchlight decoding combines the signals from a small cluster of contiguous electrodes, and scans throughout the cortical surface covered by all electrodes. Thus, the resulting searchlight decoding map retains spatial information. This allowed us to map electrode locations on the brain surface according to the maximal amount of information that they might carry, comparable to similar approaches used in functional neuroimaging (Kriegeskorte and Bandettini, 2007).

Based on their location, we grouped electrodes as belonging to either the ventral or the lateral temporal cortex (Figure 1B and C). As is typical in field potential recordings during epilepsy monitoring, precise electrode locations varied across subjects, making comparisons across subjects difficult with a conventional analysis. Our decoding analyses are powerful alternative ways to solve this problem, since they optimally blur the precise anatomical location of electrodes, which is variable from subject to subject.

### Temporal characteristics of face processing

**Figure 3:**
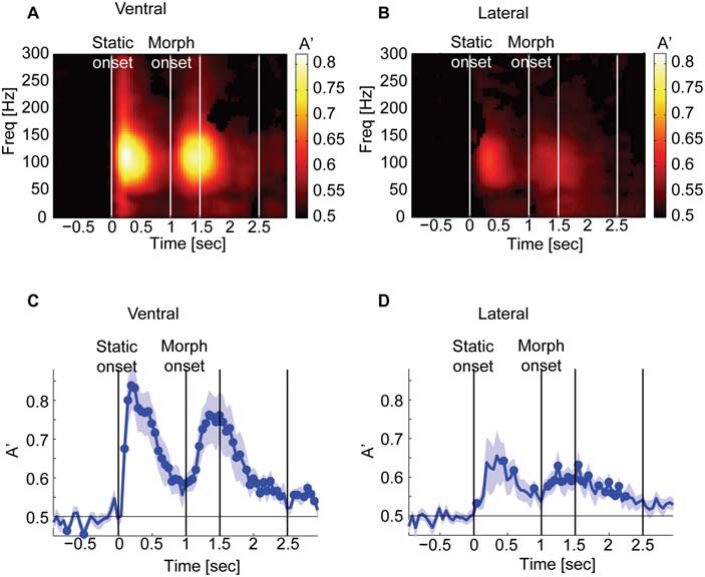
Decoding performance for face vs. checkerboard discrimination in the ventral (A and C) and the lateral (B and D) temporal cortex. A and B Time-frequency decoding map. The onset of static (*t* = 0) and morph (*t* = 1) and the offset of morph (*t* = 1.5) and static (*t* = 2.5) stimuli are indicated by vertical lines. Two peaks of decoding performance are found centered around 100 Hz. Only significant pixels are color-coded (FDR q<0.1; p<0.063 for A and p<0.054 for B). C and D Time course of decoding, combining the power across electrodes and frequencies. We marked with circles the time points where the decoding performance is significantly above chance (FDR q<0.05; p<0.035 for C and p<0.011 for D). One standard error of the mean is shown by blue shading. For this analysis we used a 100-msec window with a step size of 50 msec, with an effective frequency resolution of 20 Hz. For the ventral temporal cortex (A and C), we obtained the data from 21 sessions in 8 subjects and pooled across 23.8 electrodes (mean across sessions). For the lateral temporal cortex (B and D), we obtained the data from 22 sessions in 9 subjects and pooled across 79.1 electrodes.

First, we combined the logarithm of the event-related power from all electrodes, separately for the ventral and the lateral temporal cortex, and computed the time-frequency decoding map to characterize the critical time-frequency points for face processing. For face vs. checkerboard discrimination, decoding performance in the ventral temporal cortex was very high, with most information contained in a frequency band of 50–150 Hz (Figure 3A) shortly after the stimulus onset (0.1–0.5 sec had A' = 0.84–0.86) as well as after the onset of morphing epoch (1–2 sec had A' = 0.75–0.80). Decoding performance in the lateral temporal cortex was lower, and while most information was similarly contained in a band of 50–150 Hz after the onset of the static stimulus (A' = 0.65), it was represented more broadband (0–150 Hz) after the onset of the morphing epoch (A' = 0.63–0.67; Figure 3B).

By combining all frequencies across electrodes, we further characterized the decoding time course to obtain decoding latency and maximum decoding accuracy (Figure 3C and D, Table 1). The ventral temporal cortex showed higher decoding accuracy than the lateral temporal cortex throughout the stimulus presentation, including the morphing epoch. The emotional facial movement evoked activity related to discrimination of faces from checkerboard in both the ventral and the lateral temporal cortices, with the former carrying more information than the latter.

**Table 1:** Summary of decoding performance.

	Patients	Sessions	Electrodes	Static Face vs. Checker	Dynamic Face vs. Checker	Dynamic Happy vs Fear
Ventral temporal cortex	8	21	23.8±16.4	A' = 0.86 (87 msec)	A' = 0.80	A' = 0.61 (344 msec)
Lateral temporal cortex	9	22	79.0±15.5	A' = 0.65 (240 msec)	A' = 0.67	A' = 0.55 (946 msec)

Peak decoding performance (in A') and the latency of the decoding, defined as the first time point when the decoding became significantly above chance (FDR q>0.05).

How much of this discriminatory information was coming from the response to faces, rather than to checkerboards? Assuming that high-gamma power was correlated with local multi-unit activity [10]–[12], we examined whether the average high-gamma power (50–150 Hz, from t = 1.1–1.9 sec, during the morph period) was higher for faces than for checkerboards. For the ventral temporal cortex, compared to checkerboards, the high-gamma responses to faces were higher (t-score>3 from paired t-test, uncorrected for multiple comparisons) in 10.4% of electrodes ( = 33/316) and lower (t-score<−3) in 3.5% of electrodes ( = 11/316). For the lateral temporal cortex, the pattern was opposite; the high-gamma responses for faces were higher in 3.3% and lower in 6.7% of electrodes. The entire distribution of t-scores was significantly more positive (i.e, more electrodes showed higher responses to faces than to checkerboards) in the ventral than the lateral temporal cortex (p<1e-8, Kolmogorov-Smirnov test). While we do not claim that decoding accuracy was solely dependent on the specific high-gamma increase to faces, we conclude that decoding accuracy in the ventral temporal cortex was heavily dependent on the increased power evoked by faces.

### Ventral temporal cortex discriminates emotion more rapidly and accurately than lateral temporal cortex

**Figure 4 pone-0003892-g004:**
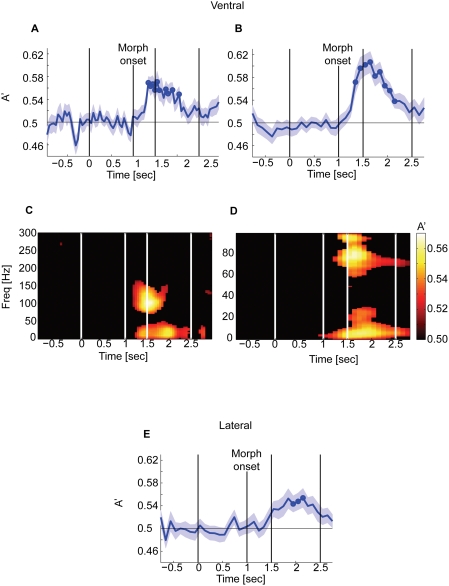
Ventral temporal cortex discriminates emotional expression more quickly and accurately than lateral temporal cortex. A–C Time course of decoding. D and E Time-frequency decoding map. A, B, D, E The results for the ventral temporal cortex and C for the lateral temporal cortex. For A and D, we used a 100-msec time window with a step size of 100 msec, giving an effective frequency resolution of 20 Hz. For B, C, and E, we used a 500-msec window with a step size of 100 msec, giving an effective frequency resolution of 4 Hz.

A dominant view of face perception proposes that regions in dorsal and lateral temporal cortex, notably the area around the STS, are specialized for processing changeable facial features that are important for social communication, including facial expressions [13], [14]. Contrary to this view, we found that emotion decoding performance was better and faster in the ventral than in the lateral temporal cortex (Figure 4).

Using a time window of 100 msec, the time-frequency decoding map for emotion discrimination reached above chance only for the ventral temporal cortex. The decoding accuracy for the ventral temporal cortex showed two peaks: one in the high gamma range and the other at lower frequencies (.3 sec to .9 sec after morph onset; FDR q<0.1, p<0.0091; Figure 4D). To resolve the two peaks in lower frequencies, we used a longer time window of 500 msec and an effective frequency resolution of 4 Hz. For this analysis, we used a step size of 100 msec and analyzed the data up to 100 Hz. With this resolution, we found one peak at the frequency below 30 Hz and the other peak above 60 Hz (q<0.1, p<0.016) (Figure 4E). The lateral temporal cortex did not show a consistent time-frequency decoding map across subjects and none of the time-frequency pixels survived the statistical threshold (FDR q>0.1).

We analyzed the time course of decoding using a short (100 msec) and a long (500 msec) time window. With the shorter time window and smaller time steps, emotion decoding performance reached above chance only in the ventral temporal cortex (peak A' = 0.57, 0.34 sec after morph onset, q<0.05, p<0.0093). With the longer window and better frequency resolution, emotion decoding accuracy reached above chance in both regions, however, it was better and faster in the ventral (peak A' = 0.61, 0.34 sec after the morph onset, q<0.05, p<0.012) than in the lateral temporal cortex (peak A' = 0.55, 0.95 sec after the morph onset, q<0.05, p<0.0066).

Taken together, our results are consistent with the hypothesis that the ventral temporal cortex performs an initial analysis of several aspects of faces, which would include diagnostic information about the categorization of the facial expression [15], [16], whereas the lateral temporal cortex appears to be more important for later stages of processing, possibly related to integration of the information across different modalities and to motor planning for social interaction [17] (see Discussion for further considerations).

### Task-relevant attention improves decoding performance across frequencies

**Figure 5:**
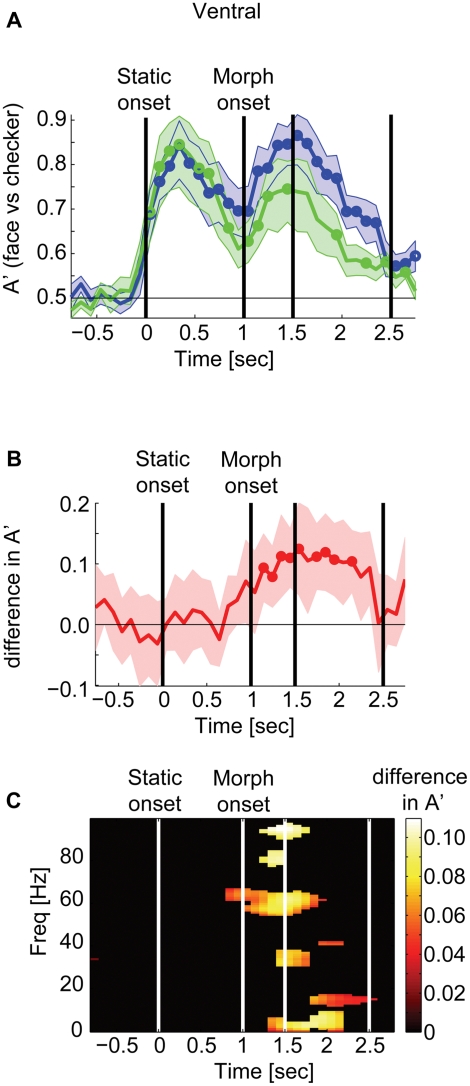
Task-relevant attention improves decoding. A Decoding performance for discriminating face vs. checkerboard (A'_fc_) when subjects were performing the emotion- (blue) or the gender- (green) task in the ventral temporal cortex. Circles mark the points where the decoding performance is significantly above chance (q<0.05, p<0.029). We combined 24.0 electrodes across all frequency bands. B The mean difference in decoding accuracy for A'_fc_ in the emotion-task minus A'_fc_ in the gender-task sessions (thick red line). The positive difference reached significance, marked by circles around the time when the stimuli started to morph (*t* = 1; q<0.05, p<0.012). The peak difference reached 11% at the maximum. Shading represents one standard error above and below the mean. C Time-frequency map for the difference in A'_fc_. The attentional effects were distributed across the frequencies. The map is thresholded at FDR q<0.1, p<0.0079. For this analysis, we used a time window of 500 msec with a step size of 100 msec, the effective frequency resolution was 4 Hz.

Decoding analyses also provide insights into the effects of task-related attentional modulation. We manipulated subjects' attention with the task instruction. In the sessions where subjects performed the emotion discrimination task, we expected they would pay more attention to faces at the beginning of the morph period because emotional expression was first revealed at that point in time. On the other hand, in the sessions where they performed the gender discrimination task, we expected they would pay more attention at the beginning of the static period. In the ventral temporal cortex, decoding accuracy for discriminating faces from checkerboard (A'_fc_) was above chance both in the emotion- and in the gender- discrimination sessions (Figure 5A) (The results shown were obtained with a 500 msec time window, but similar results were also obtained with a 100 msec window, data not shown). A'_fc_ during the morph period was significantly better in the emotion- (the peak A'_fc_ = 0.85) than in the gender- discrimination sessions (the peak A'_fc_ = 0.74); the difference (i.e., A'_fc_ [in emotion sessions]−A'_fc_ [in gender sessions]) reached significance right after the start of the morph period (*t* = 1.15 sec from the stimulus onset), attained its peak of 0.11 at *t* = 1.5 sec, and remained until the subject's button-push response (*t* = 2 sec) (Figure 5B, q<0.05, p<0.02). To examine which frequency bands are responsible for these attentional effects, we used time-frequency decoding maps and computed their A'_fc_ difference. Interestingly, the attentional effects were not localized in particular frequencies, but distributed across frequencies (Figure 5C, q<0.1, p<0.0079).

We further hypothesized that the decoding accuracy for discriminating emotion (A'_em_) and gender (A'_gn_) would be modulated by the task-instruction; A'_em_ should be higher in the emotion- than that in the gender- discrimination sessions, while A'_gn_ should be higher in the gender- than that in the emotion- discrimination sessions. However, we did not observe any such effects (See Discussion.) For the lateral temporal cortex, we did not find any significant attentional effects of any kind.

### Searchlight decoding for anatomical information

**Figure 6:**
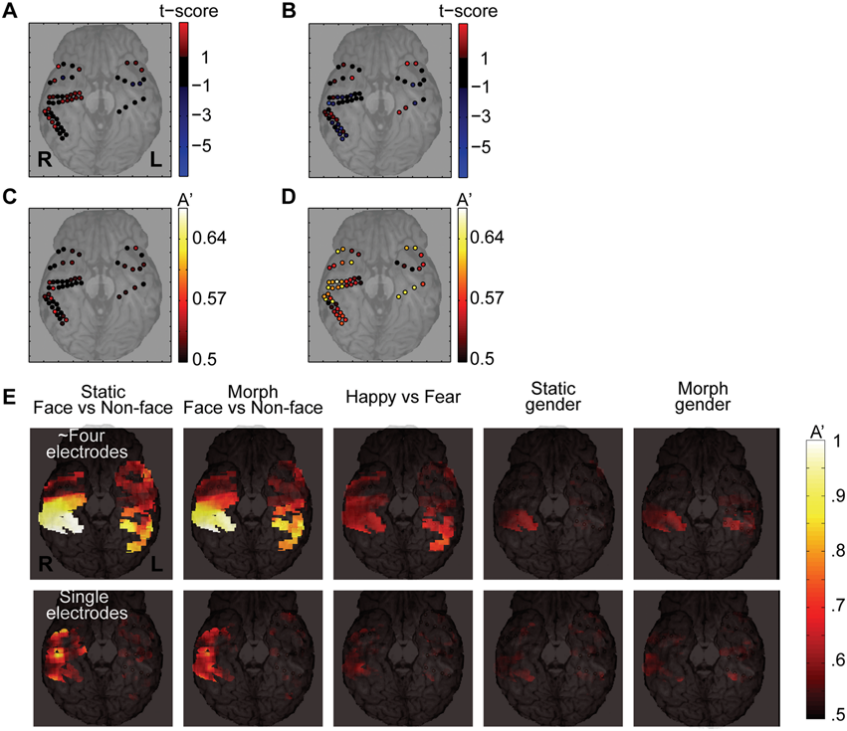
Searchlight decoding map in the ventral temporal cortex. A–D Comparison of the classic analysis (A, average power below 50 Hz; B, above 50 Hz) and decoding analysis (C, single-electrode based decoding; D, searchlight decoding). A and B The mean event-related power from 100–900 msec from the onset of the morph epoch was contrasted between the happy and the fear morphing trials. The difference was evaluated with a t-test and thresholded at |t|>1. Red color indicates greater response to happy, blue indicates greater response to fear. C Single-electrode based decoding. D Searchlight decoding for the same subject (subject 153, two sessions). Here, the spectrogram was estimated with a time window of 500 msec, with a step size of 100 msec as in Figure 5A and B. E The average decoding map across 8 subjects (see Method for how we averaged decoding maps). Decoding accuracy in the bilateral middle ventral temporal cortex improved substantially by pooling neighboring electrodes (top), compared to the near-chance decoding obtained with a single-electrode decoding analysis (bottom). For emotion decoding (middle), the pooling by searchlight improves the decoding performance substantially. For the gender decoding (right two columns), there are discriminating clusters in the middle fusiform gyri. Note the best decoding accuracy originates roughly from the same locations among five panels in the top row. Each color-coded pixel shown was covered by electrodes from at least two subjects.

To reveal the anatomical organization of face processing, we created an electrode-based decoding map. In conventional analyses, response selectivity of each electrode is mapped by averaging the power with some cutoff for the frequency band (such as below or above 50 Hz in Figure 6A and B). In our single electrode-based decoding analysis, we combined the power in each electrode optimally linearly across frequency and time (100–900 msec from the stimulus onset, Figure 6C). Combination of several neighboring electrodes within a “searchlight” improved the decoding accuracy (Figure 6D). Here, a “searchlight” is defined as a narrow circular field of view, which contains on average four neighboring electrode contacts. To create a searchlight decoding map, we scanned through the cortex by the searchlight to cover all the electrodes.

To obtain a single-electrode-based decoding map across subjects, we smoothed the decoding accuracy and then averaged across subjects (See Methods). The results for the ventral temporal cortex are shown in the bottom row of Figure 6E. The relatively poor decoding performance is expected for several reasons in addition to the general advantage in the searchlight decoding (Figure 6E, top row). First, sensitive electrodes with high decoding accuracy are often abutted by poor neighboring electrodes as can be seen in Figure 6C. Such a situation often arises when two electrodes are separated by a sulcus, reflecting an anatomical discontinuity. In this case, simple spatial smoothing degrades the performance of the best electrodes. Second, the precise locations of the best electrodes are not consistent across subjects, resulting in further deterioration of the apparent single-electrode decoding accuracy when decoding maps from multiple subjects are averaged.

Searchlight decoding solves these problems. This approach uses an optimal weighting among locally adjacent electrodes, so that the resulting map retains the anatomical information about electrode location while being more robust with respect to inter-subject variability; even if exact locations of the most sensitive electrode vary across subjects, the averaging on the searchlight decoding map does not destroy such a local peak information as in the case of the single electrode-based decoding explained above.

Searchlight decoding revealed information-carrying regions that correspond to the FFA in the ventral cortex (Figure 6E, top row) and to the STS in the lateral temporal cortex (data not shown). Discriminating faces from checkerboard pattern (A'_fc_) in the right middle fusiform gyri reached almost 100% (Figure 6E, left two columns in the top row). The apparent discrepancy between this perfect searchlight decoding and the maximal decoding performance in the time course of decoding (A'_fc_ = .85, Figure 2C) is due to sampling bias of the electrodes and the subjects in the searchlight decoding. As can be seen in Figure 1B, four subjects had electrodes roughly around this right middle fusiform region. To see if this right middle fusiform region always contains the most sensitive electrode, further studies would be needed. It is interesting to note that the right FFA-like region seems to carry information about emotion (Figure 6E, middle) and gender (Figure 6E, right two columns). To perform the appropriate statistics here, however, we would need a larger sample of subjects.

Though it is difficult to perform statistical analysis on the searchlight decoding map, we see a hint of hemispheric specialization in terms of decoding accuracy in Figure 6E. We followed up these possible laterality effects in a final analysis.

### Laterality effects

Using searchlight decoding, we had observed an apparent right hemisphere dominance for face processing, consistent with prior studies [18]–[21]. Here, we grouped electrodes in each hemisphere into three subregions; the anterior and the posterior lateral temporal cortices, and the ventral temporal cortex. Decoding was performed by combining across time and frequency in the same way as searchlight decoding.

**Figure 7:**
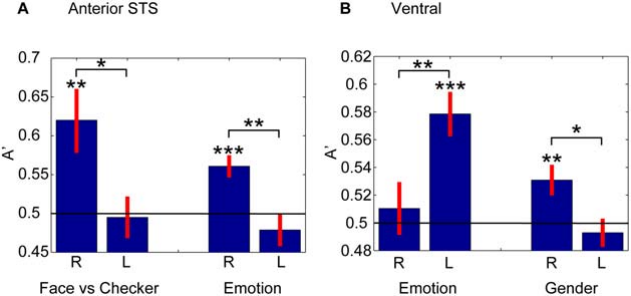
Laterality effects. A In the anterior STS region, the decoding accuracy for discrimination of face from checkerboard (A'_fc_, left) and for discriminating emotion (A'_em_, right) was better in the right than that in the left hemisphere. B In the ventral temporal cortex, a right hemisphere dominance was also found for gender decoding (A'_gn_, right). However, the left hemisphere was superior to the right hemisphere when decoding emotion (A'_em_, left). *, **, and *** indicates the significance level of p<0.05, p<0.01, and p<0.001, respectively.

We found evidence for a right hemispheric dominance in the anterior STS (Figure 7A) and the ventral temporal cortex (Figure 7B). In the anterior STS, decoding accuracy for discrimination of face vs checkerboard (A'_fc_) and emotion (A'_em_) was above chance in the right hemisphere (p<0.01 for A'_fc_, and p<0.001 for A'_em_, t-test with a null hypothesis of A' = 0.5; 11 sessions, 5 subjects, mean number of electrodes = 40.0) but not in the left hemisphere (p>0.05 for both A'_fc_ and A'_em_; 11 sessions, 4 subjects, mean number of electrodes = 38.4). The difference was significant (two-tailed unpaired t-test, p<0.05 and p<0.01 for A'_fc_ and A'_em_, respectively). In the ventral temporal cortex, decoding accuracy for discrimination of gender (A'_gn_) was above chance in the right (p<0.01, 17 sessions, 7 subjects, mean number of electrodes = 18.9) but not in the left hemisphere (p>0.05, 21 sessions, 8 subjects, mean number of electrodes = 11.0) with a significant difference (two-tailed unpaired t-test, p<0.05). Unexpectedly, in the ventral temporal cortex, emotion decoding (A'_em_) was above chance in the left (p<0.001) but not in the right hemisphere (p>0.05), with a significant difference (p<0.01). Though a right hemisphere advantage for face processing has been reported previously [18]–[21], the superior emotion processing in the left ventral temporal cortex has not (although there are reports of a left amygdala advantage [22]). Decoding analysis, however, can reveal only the information available in principle, not how and whether that information is used by the brain to guide behavioral discrimination. It is possible that diagnostic facial features that are critical for emotion detection [16] are processed automatically and represented more accurately in the left hemisphere, but that the integration of information required for behavioral discrimination is performed in the right hemisphere.

## Discussion

Considerable effort has been devoted to showing that, among other stimulus categories, faces are processed preferentially by specific anatomical structures [23]–[26] and can evoke scalp EEG and MEG responses at certain latencies [15], [27]–[33]. Yet anatomical space and detailed processing time have generally not been mapped jointly, in large part because doing so requires rarely available methods such as the intracranial recordings we present here [1]–[4], [28], [34], [35]. We analyzed the intracranial ECoG with a decoding technique and found that 1) the best discrimination of faces from checkerboards arose within a critical frequency band of 50–150 Hz in the ventral temporal cortex, 2) this held for both static and dynamic stimuli, 3) the accuracy of decoding was much better in ventral as compared to lateral temporal cortex, for faces vs. checkerboards, and also for happiness vs. fear, 4) in the ventral temporal cortex, task-relevant attention improved the decoding accuracy for stimulus category (A'_fc_) across wide frequencies by as much as 11%, but it did not improve decoding accuracy for emotion (A'_em_) and gender (A'_gn_), and 5) the anterior STS and the ventral temporal cortex showed evidence for hemispheric specialization of face processing.

### Role of ventral and lateral temporal cortex in face processing

An influential model of face processing [36] hypothesized that face identity and emotional expression are processed by functionally separate systems. More recently, this idea has been resurrected on the basis of findings from cognitive neuroscience: invariant features of faces (i.e., identity) appear to be processed predominantly in ventral temporal cortex, including the fusiform face area (FFA) [24], [37], while changeable features of faces, such as emotional expression, appear to be processed in lateral temporal cortex, including the superior temporal sulcus (STS) [13], [14]. This popular theory of face processing has been supported by neuropsychological studies of subjects with focal lesions, as well as by fMRI and single cell physiology (For reviews, see [13], [36]). However, a careful review of the literature casts some doubt on the extent to which processing of face identity and expression are truly independent. For instance, while there are prosopagnosic subjects with severely impaired identity recognition yet spared emotion recognition, there is no known case of severely impaired general recognition of emotions with spared recognition identity (although there are cases with selective impairments in recognizing certain specific emotions [38], [39]). Whether identity and emotion information are processed by entirely separate neural structures is still open to debate [17].

We found that decoding performance in the ventral temporal cortex around the fusiform gyrus was much superior to the lateral temporal cortex, including the STS. The ventral superiority was expected during the static period; surprisingly, however, this held true for discrimination of faces from checkerboards during the morph period as well (A'_fc_ at t = 1–2 sec). In our paradigm, the identity of a face is revealed to subjects at the onset of a trial and remains constant throughout the trial, in particular, it is constant during the morph period. Thus, no additional information relevant to face vs. checkerboard discrimination is revealed during the morph period. Decoding performance A'_fc_ in the ventral temporal cortex peaked immediately after the stimulus onset (A'_fc_ = 0.85), and after it fell below A'_fc_<0.6, it quickly improved at the onset of morphing (A'_fc_ = 0.77, Figure 3). Our analysis based on the average high-gamma activity during the morph period showed that many electrodes in the ventral temporal cortex increased activity to faces, while those in the lateral temporal cortex increased activity to checkerboard patterns. This pattern of results strengthens the idea that the ventral temporal cortex serves as a general ‘face processor’, which responds to facial movements, even without any change in identity. While we cannot rule out the possibility that motion of the stimulus strongly attracted attention and therefore activated the ventral temporal cortex, we note that such facial motion would be expected to attract more attention in the lateral than the ventral temporal cortex according to the standard view. Our results were not consistent with what that theory would predict. Further studies will be needed to investigate whether the same ventral temporal region also responds to other types of biological motion, such as gaze shifts or movements of the mouth during speech.

Better and faster emotion decoding accuracy (A'_em_) in the ventral than the lateral temporal cortex indicates that information processed within the ventral cortex at early latencies might contain critical and sufficient information to discriminate emotional expressions. It is plausible that the activation in the ventral temporal cortex reflects automatic processing of some facial features such as eyes [15], diagnostic for certain emotion categorizations [16], but may not be causally related to the recognition of emotional expression, which can be supported by regions other than the ventral temporal cortex (Adolphs, 2002).

The worse decoding accuracy for emotion (A'_em_) in the lateral than the ventral temporal cortex might be due to other reasons. It is possible that the collective neuronal activity measured by our field potential recordings may have smeared out more fine-grained encoding of emotional expression information at the level of single neurons [40], [41], and that this effect may have differentially affected the regions around the putative FFA and STS. Such spatiotemporally fine-grained information would not have been detected by individual surface electrodes because the detectable information reflects the integrated activity at the level of a neuronal population. Another potential reason may be that our surface electrodes were less sensitive to information from cortex buried within sulcal folds, as they are for information from subcortical structures with non-uniform dendritic arborization [42]. If an electrode were buried in the sulcus of the lateral temporal cortex, emotion decoding performance might improve substantially, and possibly better than for the ventral temporal cortex.

### Rapid categorization in ventral temporal cortex?

We found that field potentials recorded from the ventral temporal cortex discriminate faces from checkerboards rapidly. Previous EEG [28]–[31], [43], [44] and MEG studies [32] found an early evoked potential before or around 100 msec that is correlated with stimulus categorization. This rapid categorization may not reflect subjects' decisions to categorize the stimulus, but rather statistical image properties of the different categories of the stimuli [43]. Alternatively, this rapid response may be correlated with behavioral categorization, especially for categorization of stimuli as face vs. non-face objects [32]. Because we were originally motivated to study the neuronal response during dynamically morphing facial expressions, our choice of control stimuli (high-contrast checkerboards) was not optimal to study rapid categorization of objects at such a short latency. We are now addressing these questions and extending our current findings by using other classes of stimuli. Can rapid response in the ventral cortex categorize several classes of objects?

We note that the exact relationship between the rapid power modulation in high-frequency bands that we observed and the early component reported in EEG and MEG previously is unclear. The early EEG/MEG component is dominated by the stimulus-locked (i.e., response phase is constant across trials) power modulation in low frequency bands with minimal contribution from high frequency bands because the power spectrum of the electroencephalogram has a 1/f distribution (Figures S1 and S2 C–E). Our decoding was mainly based on power modulation in high-frequency bands (e.g., Figure 3). An interaction between low and high frequency responses was demonstrated in a recent study [45] that found robust coupling between the phase of the theta rhythm (4–8 Hz) and the power of high gamma responses. Further studies using a stimulus-evoked response paradigm will be required to reveal the relationship between the transient power increase we found in high-frequency bands and its power modulation by the phase of lower frequency oscillations.

### Attentional improvement of decoding performance

Depending on the task instruction, the decoding accuracy for discriminating faces from checkerboards (A'_fc_) improved by 11% in the ventral temporal cortex. During the morph period, attention to facial emotion improved A'_fc_ across wide frequencies compared to attention to facial gender (Figure 6C).

Task-relevant top-down attention is known to modulate neuronal firing rate [46], [47], event-related power in the high gamma range [48] and BOLD fMRI signals [49]. Recently, attention has been shown to improve decoding performance in fMRI [50]. Another potential effect of attention is the modulation of communication between separate cortical regions. Recent neurophysiological studies [51], [52] examined the role of coherence between spikes and local field potentials and showed specific increases in spike-field coherence in the gamma range (∼40 Hz), together with decreases in the beta range (∼20 Hz). Our differential time-frequency decoding map (Figure 7C), however, revealed rather distributed effects of attention across frequencies. It is worth noting that the peak decoding difference in the time-frequency map (Figure 5C) was comparable to the peak decoding time course (Figure 5B), implying no advantage in combining the attentional effects across frequencies. In other words, the attentional effects may be present in broadband, but highly correlated across frequencies. We also note that if attention were to change cross-frequency coherence to improve inter-areal communication (e.g., via modulating signal/noise correlation [53]), the attentional effects for decoding across frequencies and channels (Figure 5B) would be higher than the attentional effects for decoding across channels at each time-frequency (Figure 5C), which was not the case. This type of effect is expected if the power of the field potential is modulated uniformly across frequencies. It is tempting to suggest that our observed effects may reflect an increase in firing rate without specific oscillatory components.

### Lack of attentional effects on emotion & gender discrimination

Though attention improved the decoding accuracy for faces vs. checkerboards (A'_fc_), it did not modulate the decoding accuracy for emotion (A'_em_) or gender (A'_gn_) during either the static or morph periods. The lack of attentional modulation for gender decoding (A'_gn_) may be due to floor effects; we did not find good decoding accuracy for gender discrimination in any recording location (Figure 6), time window, or frequency band. Another possible reason is that our gender task was too easy to engage any attentional effects (behavioral accuracy was 95% correct). In fact, the task can be performed by seeing the stimulus only briefly at any time point during the 2.5 seconds of stimulus presentation, possibly resulting in a temporal spread of attentional effects that are inconsistent across trials and subjects.

By contrast to gender discrimination, emotion discrimination required more temporally focal attention, especially around the onset of the morph epoch, improving the decoding of faces vs. checkerboards (A'_fc_) (Figure 5). As the emotion decoding (A'_em_) was above chance in both the ventral and the lateral temporal cortex (Figure 4), the lack of attentional effects are unlikely to be due to floor effects. Again, the task might have been too easy to observe any attentional effects (behavioral A' was 0.90 for emotion discrimination). Using five different categories of emotional faces (which is presumably more attentionally demanding than our stimulus set), Krolak-Salmon et al (2002) reported strong attentional effects of emotion processing in event-related potentials recorded intracranially from the amygdala. Future studies utilizing more demanding tasks or more emotion categories would be necessary to reveal the nature of attentional modulation in the ventral temporal cortex.

### Value of the decoding approach for intracranial EEG

The direct advantages of decoding for intracranial EEG are three-fold: 1) It allows visualization and provides a concise summary of high dimensional data, and is thus especially well-suited for time-frequency analyses of multi-channel recordings; 2) It avoids severe multiple comparison problems inherent to multi-channel time-frequency analyses, which often lead to a rather arbitrary selection of a set of particular electrodes, particular frequency bands, and particular time ranges to be statistically evaluated; 3) It facilitates averaging of data from multiple subjects. In intracranial recordings, electrode distributions as well as the exact locations of sensitive electrodes vary across subjects. With searchlight decoding we combined neighboring electrodes, and with the time-frequency decoding map and time course of decoding we combined all the electrodes within a larger anatomical unit. These operations optimally and linearly blurred fine anatomical structures, making the decoding performance comparable across subjects despite their inhomogeneity. We believe this is an alternative powerful analysis method, useful for future studies, when one is interested in questions at the system level with precise time-frequency resolution.

Those three benefits of the decoding approach are interrelated. We provided an example of the classical analysis in Figure 6A and B, where these problems can be easily appreciated. To present a spatial map of the time-frequency response, a considerable amount of information gained from the time-frequency analysis is simply wasted due to averaging across time and frequency. In Figure 6A and B, we summed the evoked power above or below an arbitrary frequency (i.e., 50 Hz), but this is clearly not the optimal strategy. Even if one finds an optimal selection of frequency bands, time points, and spatial locations for averaging, this selection tends to ‘over-fit’ to a particular data set, which generalizes poorly to different subjects. We overcame this problem by optimally linearly combining the response along frequency and time for each subject with an objective and automatic decoding procedure and evaluating the decoder's performance on the test trials, which the decoder did not see during training. Although we lost some spatial specificity (including the polarity of the response only visible in Figure 6A and B), combining electrodes ‘blurred’ fine spatial structure optimally and linearly and permitted pooling across subjects. A similar problem arises in high-resolution fMRI, where fine spatial patterns of the response make it difficult to average across subjects [54], [55]. In other words, in both intracranial ECoG and high-resolution fMRI, the spatial resolution is much finer than the spatial jitter inherent to individual anatomical differences. If simple smoothing were used, the very advantage conferred by high spatial resolution is totally discarded. Even though spatial specificity of the response is best preserved in the raw data for each individual subject, we cannot generalize and replicate such a finding to other individuals; we therefore opted for better averaging across subjects at the expense of too fine spatial resolution. The same problems arise for the high temporal resolution of the ECoG. The very advantage of high time-frequency resolution is wasted if one simply averages across time and frequency. The decoding technique on which we capitalized in our study is a powerful alternative for analyzing multi-channel field potentials across individuals by preserving high spatio-temporal resolution with minimal assumptions about timing, frequency and spatial locations of interest.

Finally, we point out the general advantage of decoding analyses: decoding performance (such as A' = 0.8 or 80% correct classification) is intuitive and objective. Compared to decoding analyses, conventional statistical analyses can be difficult to interpret because many factors affect the resulting estimates of significance (e.g., whether or not the assumptions of the response distribution are met, how many subjects and trials are tested, whether there were correlations among the data, etc). This is especially true when multiple factors are considered in a multivariate analysis, where one can easily over-fit the data. Decoding analyses prevent over-fitting with a cross validation procedure (i.e., separate training and test trials) and offers a very intuitive ‘accuracy’ measure, such as % correct or A'. Modern sensory neurophysiology, for example, compares different models of neuronal response within a decoding framework [56]. As the analysis of electrophysiological data becomes more sophisticated, the intuitive and objective nature of a decoding approach becomes increasingly important. For example, we might be able to improve decoding performance by devising an optimal exclusion criterion for trials and/or electrodes. Similarly, we could objectively compare different kinds of preprocessing techniques, such as source modeling and independent component analysis, and quantify the degree of improvement afforded by each of these. Decoding analyses not only facilitate comparisons across different neuronal measures (such as EEG, MEG, fMRI), physiological measures (such as eye movements, skin conductance), and different aspects of a particular measure (such as the event-related power, the phase, and the degree of synchrony of multiple ECoG) but they ultimately allow us to combine these measures to provide the best inference of our mental life from a third-person perspective.

## Materials and Methods

### Subjects

We obtained written informed consent from nine patients with medically intractable epilepsy (see Table 2 for detailed demographic information) who were undergoing epilepsy monitoring to guide neurosurgical treatment. The study was approved by the Internal Review Board at the University of Iowa. The patients underwent electrode implantation under a purely clinical protocol and the location of electrodes was determined solely by medical considerations. All patients were on anticonvulsant medications in reduced or absent dosage to facilitate the occurrence of seizures to aid in the clinical detection of the seizure foci. The experiments reported here were conducted typically 6–10 days after the implantation of the electrodes. Recording sessions were kept as brief as possible and were dependent on the patient's willingness for research participation at a given moment as well as on clinical constraints. We did not record when our experiments introduced any clinical inconvenience, and we did not record for 24 hours after any major seizure.

**Table 2:** Patients' demographic information.

ID	Age	Sex	Education	Handedness	Language	Side of Grid	Seizure focus
138	20	M	14	R	L	L	Left anterior lateral temporal, independent right mesial temporal interictal discharge
139	53	F	12	L	R	R	Right mesial temporal
140	26	M	10	R	L	L	Left anterior lower parietal
142	33	F	12	R	L	R	Right mesial temporal
146	29	F	16	R	L	L	Left mesial temporal
147	29	M	14	L	L	L	Left posterior ventral temporal cortex
149	22	M	11	L	Bilateral	R	Bilateral mesial temporal
153	31	F	15	R	L	R	Right mesial temporal
154	40	M	13	R	L	R	Right mesial temporal

Education is indicated in years.

### Anatomical location of the electrodes

On the lateral temporal cortex, all nine subjects had grid electrodes with 64–96 contacts (mean across subjects = 77.6, std 16.3); five had them on the right hemisphere, and four on the left. Inter-electrode distance of the lateral temporal grid was 5 mm. The grids were configured in a rectangular matrix of 4×8, 8×8, or 12×8. The location of the grid was roughly similar across subjects (Figure 1C). On the ventral temporal cortex, eight subjects had several strip electrodes in each hemisphere: seven subjects had 4–40 contacts (mean 16.5, std 17.5) in the right hemisphere and eight subjects had 4–16 contacts (mean 11.0, std 4.7) in the left hemisphere. The location and number of the strip electrodes varied (Figure 1B). Ventral electrodes were either 4-contact strip electrodes or 2×8-contact strip-grid electrodes. Inter-electrode distance of the 4 contact strip electrode was 1 cm and that of 2×8 strip electrode was 5 mm.

For each subject, we obtained structural T1-weighted MRI volumes (pre- and post- electrode implantation), CT scans (post-implantation) and digital photos of the electrodes (during surgery, only for the lateral temporal grid electrodes). Coronal slices of the MRI were obtained with 1 mm slice thickness, 0.78×0.78 mm in-plane resolution. Axial slices of the CT scans were obtained with 1 mm slice thickness, 0.45×0.45 mm in-plane resolution. Post-implantation CT scans and pre-implantation MRI were rendered into 3D volumes and co-registered using AFNI (NIMH, Bethesda, MD, USA) and ANALYZE software (version 7.0, AnalyzeDirect, KS, USA) with mutual information maximization.

Because the ventral temporal strip electrodes were not directly visible during surgery, we did not take any digital photographs of them. However, as the contacts on the strip electrodes were not as dense as in the lateral temporal grids, post-implantation CT scans were sufficient to identify the coordinates of the contacts. We transferred these coordinates onto the higher resolution pre-operative MRI for visualization purposes.

For the lateral temporal grid electrodes, the electrodes were denser than those on the strip electrodes. Therefore, after CT-MRI coregistration, we further refined the estimated coordinates of each contact by visually matching the gyral-sulcal pattern of the MRI-based surface rendering with that of digital photographs taken during electrode placement and removal surgeries.

After the locations of electrode contacts were visualized on the 3D anatomical MRI rendering, we obtained 2D projections of the MRI from ventral (Figure 1B) and lateral (Figure 1C) views, using in-house programs in MATLAB 7 (Mathworks, MA, USA). Next, we aligned the 2D projection across subjects by translation, rotation and scaling, using the transparent layers in Adobe Photoshop. For the ventral view, we aligned the outlines of the brains for each subject into that of a reference subject whose brain outline is shown in Figure 1B. For the lateral view, we first flipped the side for the right hemisphere. Then we aligned each brain into the target brain shown in Figure 1C. With translation, rotation, and scaling, we aligned the conspicuous anatomical landmarks around the temporal surface, including the lateral sulcus, the superior temporal sulcus, and the outline of the inferior frontal lobe and the anterior temporal lobe.

### Electrode density map and searchlight decoding map

We obtained an electrode density (ED) map for each subject by the following equation;
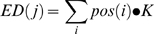
where *ED(j)* refers to an electrode density map for *j*-th subject, *pos(i)* is a 2D delta function that is zero except at the coordinate [x,y] for an electrode *i*, *i* spans across all the electrodes for *j*-th subject, •• denotes convolution, and *K* is a 2D Gaussian kernel whose full-width-at-half-maximum was the average inter-electrode distance on the 2D projection and whose extent was circular with an radius being the inter-electrode distance.

When averaging the ED maps across subjects (Figure 1D and E), we used the following equation;
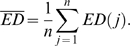
Thus, the summed pixel values in the average density map for all subjects equal the average number of electrodes across subjects.

We obtained a searchlight decoding map for one subject by the following equation;
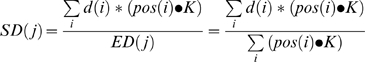
where *SD(j)* refers to a searchlight decoding map for *j*-th subject, *d(i)* is a scalar constant representing the decoding accuracy at the *i*-th electrode (or *i*-th search light) and *pos(i)* is a 2D delta function that is zero except at the center position of the *i*-th electrode (or *i*-th searchlight). We normalized the summed decoding accuracy by *ED(j)* at each pixel. If there is only one electrode (*i* = 1), *SD(j) is d(1)* for the extent of *K* and not defined elsewhere. When there is an overlap between more than two electrodes, *SD(j)* linearly interpolates the decoding accuracy.

We obtained a searchlight decoding map across subjects (Figure 6E, top row), by the following equation;
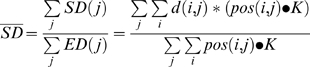



### Electrophysiological recording and stimulus display

ECoG was recorded with intracranially implanted electrodes (Ad-Tech Medical Instrument Corp., WI, USA). Electrical potential at each electrode was referenced to the electrode placed under the scalp near the vertex of the skull. The impedances of the electrodes were 5 k–20 k Ohm. Signals from the brain were digitized and recorded using the Multi-Channel Neurophysiology Workstation (Tucker-Davis Technologies, FL, USA) and analyzed offline using custom programs in MATLAB. For an initial six subjects, we used an LCD display (Multisync LCD 1760V, NEC, Tokyo, Japan) for stimulus presentation and recorded the electrophysiological signal at a sampling rate of 1 kHz. For the three latest subjects, we used another LCD display (VX922, ViewSonic, CA, USA) and recorded the signal at 2 kHz. In both cases, the display refresh rate was 60 Hz. We measured the precise timing of the stimulus onsets by presenting a small white rectangle on the top-left corner of the display and recorded the response of a photodiode directly attached at that corner. The output from the photodiode was recorded along with the electrophysiological responses in the same recording system.

### Stimuli and Task

We used gray scale pictures of neutral, happy and fearful expressions of 4 individuals (2 female) from the Ekman and Friesen set [57]. Each face was equated for size, brightness, contrast, and position and framed in an elliptical window using MATLAB. The faces subtended about 7.5×10 deg in visual angle. For the morph movie period of our stimuli, we created 28 images by linear interpolation between neutral and emotional faces (Morph 2.5, Gryphon Software, CA, USA). We presented the stimuli using Psychtoolbox [58] and MATLAB 5.2 on a Power Mac G4 running OS 9.

A trial began with a baseline static plaid pattern for 1 second, followed either by a static neutral face or by a radial checkerboard pattern (with black/white square wave modulation at around 12 cycles per face, Figure 1A). After a further 1 sec (2 seconds total from the trial onset), the static neutral face started to morph into either a fearful or a happy expression, or the radial checkerboard pattern started to expand or contract. The morph period lasted 500 msec. The last frame in the morph movie stayed on for another 1 second. After the stimulus was extinguished, subjects were prompted to make a response to discriminate the stimulus. In a given session, subjects were instructed to discriminate either the emotion or the gender of the face if they saw a face. They were asked to answer ‘other’ if they saw a checkerboard in all sessions. The prompt reminded subjects of the three alternatives as [1, happy; 2, other; 3 fear] in the emotion discrimination sessions and [1, woman; 2, other; 3, man] in the gender discrimination sessions. After the response, the next trial started. We did not put any time constraint on the response time and did not instruct subjects to put any priority over the speed or accuracy.

One session consisted of 200 trials; 80 trials of neutral-to-fearful face morphs (20 for each identity), 80 trials of neutral-to-happy morphs, 40 trials of checkerboard (20 expanding, 20 contracting).

Subject 139 and 146 performed only one session of the emotion discrimination task (subject 146 completed only 55 trials due to a technical problem). We collected electrophysiological data from a total of 22 sessions across subjects, and behavioral responses from a total of 19 sessions (3800 trials).

### Spectrogram analysis

The spectrogram (time resolved Fourier Transform) of the raw EEG signal was estimated using a multi-taper method (Figure S1 and S2, C–E) with a sliding short time window. We used three Slepian data tapers. For decoding analysis, the logarithm of the power spectrum during the baseline period (−1 to −0.5 sec from the onset of the static baseline stimulus) was subtracted to obtain the event-related power at each frequency in each trial. Input to the decoder was, therefore, the log-transformed event-related power. For the analysis of the high-gamma frequency range (up to 300 Hz), we used a time window of 100 msec with a step size of 50 msec. This gave us a half bandwidth (*W*) of 20 Hz: *W* = (*K*+1)/2*T*, with *K* being the number of data tapers, *K* = 3, and *T* being the length of the time window, *T* = 100 msec. For a time window of 500 msec with a step size of 100 msec, a half bandwidth was 4 Hz.

### Decoding procedure (General)

Using a decoding technique, we discriminated face vs. checkerboard, happy vs. fear and male vs. female by combining the event-related power in different ways; the power at each time-frequency point was combined across electrodes in the ventral or lateral temporal cortex for *time-frequency decoding*, (Figure 2 top, Figure 3A and B), across frequencies and electrodes in a given region (e.g., the lateral temporal cortex) for *decoding time course* (Figure 2 bottom, Figure 3C and D), and across times and frequencies of a few adjacent electrodes for *searchlight decoding* (Figure 6). Optimal weights (*w*) were estimated by a regularized least square classifier [7], [8]. Decoding analysis was separately performed for each session in each subject. 70% of trials were randomly selected to train the classifier, and the remaining 30% of trials were used as test trials to evaluate classification performance. In order to minimize the bias of the classifier, we sampled the same number of trials for either class (e.g., face vs. checkerboard); if the numbers of trials differed between the classes (for example, n_1_ trials for class 1 and n_2_ trials for class 2, where n_1_>n_2_), we first randomly sampled a subset of trials from the class with more trials (i.e., we randomly sampled n_2_ trials from class 1) so that the two classes had the same number of trials. This procedure resulted in n_1_ = n_2_ = 40 for face vs. checker classifier and n_1_ = n_2_ = 80 for emotion or gender classifier. We applied this general rule to one exceptional session where we had only 55 trials in total. Because we balanced the number of trials for each classes, we did not observe any decoding bias, as is seen for decoding time course in Figure 3–5; A' was at the chance level ( = 0.5) during the baseline period (t<0) in face vs. checkerboard discrimination (Figure 3C and D and Figure 5A) and before the morph period (t<1) in emotion discrimination (Figure 4A, B, and E). When discriminating face vs. checkerboard, we pooled all face trials across different emotions, genders and identity and discriminated those trials from checkerboard trials (collapsing across contraction and expansion epochs). As a result, in an extreme case, a face vs. checkerboard classifier might have been trained on 28 female happy faces and 20 contracting and 8 expanding motion trials (i.e., 70% of n_1_ = n_2_ = 40 trials is 28 trials) and then tested on 12 male fear faces and 12 expanding motion trials, a more strict test for generalization.

We assessed decoding performance using signal detection theory [9]; we sorted the output from the classifier on the test trials and subjected it to an ROC analysis. We report the non-parametric estimates of sensitivity, area under the ROC curve (A') as an index of decoding performance rather than % correct classification, because A' incorporates information in the magnitude of classifier outputs, which is totally discarded in % correct classification. We repeated the above procedure 10 times to obtain an estimate of A'. A separate classifier was trained and A' was estimated for each classifier at each data point. For example, the time-frequency decoding of emotion shown in Figure 2 (top panel), used 36360 independent classifiers (36 time points×101 frequencies; at each time-frequency point, 10 classifiers were trained with a different set of 70% of trials for training). This was repeated for each of 22 sessions.

Any abnormal trials (i.e., due to apparent epileptic spikes) did not significantly affect our decoding analysis. The aberrant trials in the training set would contribute minimally to the learning of optimal weights because we used a regularized classifier to reduce the effects of outliers [7]. Those in the test set could only reduce, not improve, the decoding accuracy. Similarly, bad electrodes or electrodes close to epileptic foci would be expected to affect our analysis only minimally because, during training, those electrodes would automatically be assigned lower weights to improve the decoding accuracy. In other words, our decoding approach automatically and objectively pruned the influence of abnormal trials and electrodes, without relying on any subjective criterion for removal of a subset of trials and electrodes (e.g., apparently large amplitude or apparent epileptic spikes).

When we averaged A's across subjects, we first converted A' into z-scores using a logit transform to normalize the distribution. All statistical tests, except for the paired t-test to quantify the attentional effects (see below), were done on the z-scores. For visualization of results, we transformed the mean and mean±one standard error of the z-scores back to A' with an inverse logit transform.

### Time-frequency decoding

Optimally combining event-related power at each time-frequency point across many channels in a circumscribed anatomical structure, we characterized information at each time-frequency point without any prior assumptions about particular frequency bands and particular time points (Figure 2, top). For the statistical analysis, we smoothed the decoding map for each subject with a 2D Gaussian kernel in time and frequency (5×5 pixels, with std = 2 pixels) then averaged across subjects. We calculated p-values using two-tailed t-tests against chance (z(A') = 0 or A' = 0.5). We corrected for multiple comparisons using false discovery rate corrections (FDR q<0.1) [59].

### Time course of decoding

Optimally combining event-related power across frequencies and channels, we characterized information in time in each anatomical region (Figure 2, bottom). No smoothing was applied in the time dimension to accurately estimate the latency of decoding. P-values from two-tailed t-tests against chance (A' = 0.5) were calculated and corrected for multiple comparisons by FDR (q<0.05). The first time point when the decoding became significant was defined as the latency of decoding (Table 1).

### Attention effects

We analyzed the effects of task-relevant attention by comparing the decoding accuracy between the emotion- and the gender- discrimination sessions. In Figure 5B, for example, we subtracted the decoding accuracy for discriminating face vs. checkerboard (A'_fc_) in sessions when subjects discriminated the gender from that when they discriminated the emotion for each subject. For this analysis, we used the raw A' for subtraction and performed the paired t-test at each time (Figure 5B) or time-frequency point (Figure 5C), because the difference of the raw A' was normally distributed.

4 subjects performed each task once and 3 subjects performed each task twice, once in the uni-directional and once in the bi-directional morph condition. In total, 10 sessions of the emotion task were paired with 10 sessions of the gender task in each subject, equated in the morph direction condition.

### Single-electrode decoding and searchlight decoding

For single-electrode and searchlight decoding (Figure 6), we combined event-related power during the static period (100–900 msec after the onset of the static stimuli) or the morph period (100–900 msec after the onset of the morphing). To roughly equate the number of inputs to the classifier, we downsampled the event-related power along the frequency dimension by 1/6 for searchlight decoding.

For the lateral temporal grid contacts, the inter-electrode distance was uniform. Thus we used a searchlight with a radius of approximately 5 mm, which covered 4 neighboring electrodes for all subjects. For the ventral lateral cortex, electrodes were placed differently for each subject. In order to retain regional specificity, we used a fixed radius, which was 1/10 of the diameter of the cerebral hemisphere. This radius contained 4 electrodes on average.

### Laterality analysis

For the laterality analysis (Figure 7), we combined event-related power along frequencies, time (100–900 msec from the onset of the morph period) and electrodes within each hemispheric region. The electrodes were grouped either in the anterior or posterior half of the lateral temporal cortex, and the left or the right hemisphere of the ventral temporal cortex. We downsampled the event-related power along the frequency dimension by 1/6.

## Supporting Information

Figure S1 Standard ERP and spectrogram analysis (1) A–H) Analysis of channel 75 for subject 153 (session 1). A ERP analysis. The average traces of field potentials are shown for fearful faces (80 trials, thick blue), happy faces (80 trials, thick red) and checkerboards (40 trials, thin black). This electrode showed large positive potential to faces at around 200 msec from the onset of the stimuli (t = 0 sec), but no such clear peak around the onset of the dynamic morph (t = 1 sec). B T-score for the difference between face and checkerboard (red) and between fearful and happy faces (blue). C–E Mean time-frequency spectrogram for fearful (C), happy (D) and checkerboard (E) conditions. Increased power around 100 Hz is seen at just after the onset of both static (t = 0 sec) and morph (t = 1 sec) period for faces (C and D) but not for checkerboards (E). Mean of the spectrogram for each trial is color-coded in log-scale. See the bar at the right for color scale. F and G T-scores for the difference between faces and checkerboards (F) and between fearful and happy faces (G), showing strong difference between conditions in high frequencies, which was not evident in the ERP analysis (A and B). See the bar at the right for color scale. H Relative power increase in the high-gamma bands (50–150 Hz). Mean high gamma power for fearful (blue), happy (red) and checkerboard (black) conditions are plotted, with the shades indicating one standard error of the mean across trials. The high-gamma power for this electrode increased relative to the baseline to faces, but not to checkerboard, at the onset of the static period. During the morph period, it increased even higher for fearful than happy faces.
(3.21 MB TIF)

Figure S2 Standard ERP and spectrogram analysis (2) A–H) Analysis of channel 74 for subject 153 (session 1). A ERP analysis. This electrode showed much larger negative and positive potential to faces at around 200 msec from the onset of the stimuli than channel 75. B T-score for the difference between face and checkerboard (red) and between fearful and happy faces (blue). C–E Mean time-frequency spectrogram for fearful (C), happy (D) and checkerboard (E) conditions. (E). Mean of the spectrogram. (F and G) T-scores for the difference between faces and checkerboards (F) and between fearful and happy faces (G). See the bar at the right for color scale. H Relative power increase in the high-gamma bands (50–150 Hz). Mean high gamma power for fearful (blue), happy (red) and checkerboard (black) conditions are plotted, with the shades indicating one standard error of the mean across trials.
(3.43 MB TIF)

Figure S3 Electrode 74 and 75 are marked by green and blue circles, respectively, in the right ventral temporal cortex.
(8.22 MB TIF)
