# Supplementary figures and images for: Decoding Face Information in Time, Frequency and Space from Direct Intracranial Recordings of the Human Brain
Source: PLoS One. 2008 Dec 9;3(12):e3892. doi: 10.1371/journal.pone.0003892 (PMC2588533; doi:10.1371/journal.pone.0003892)

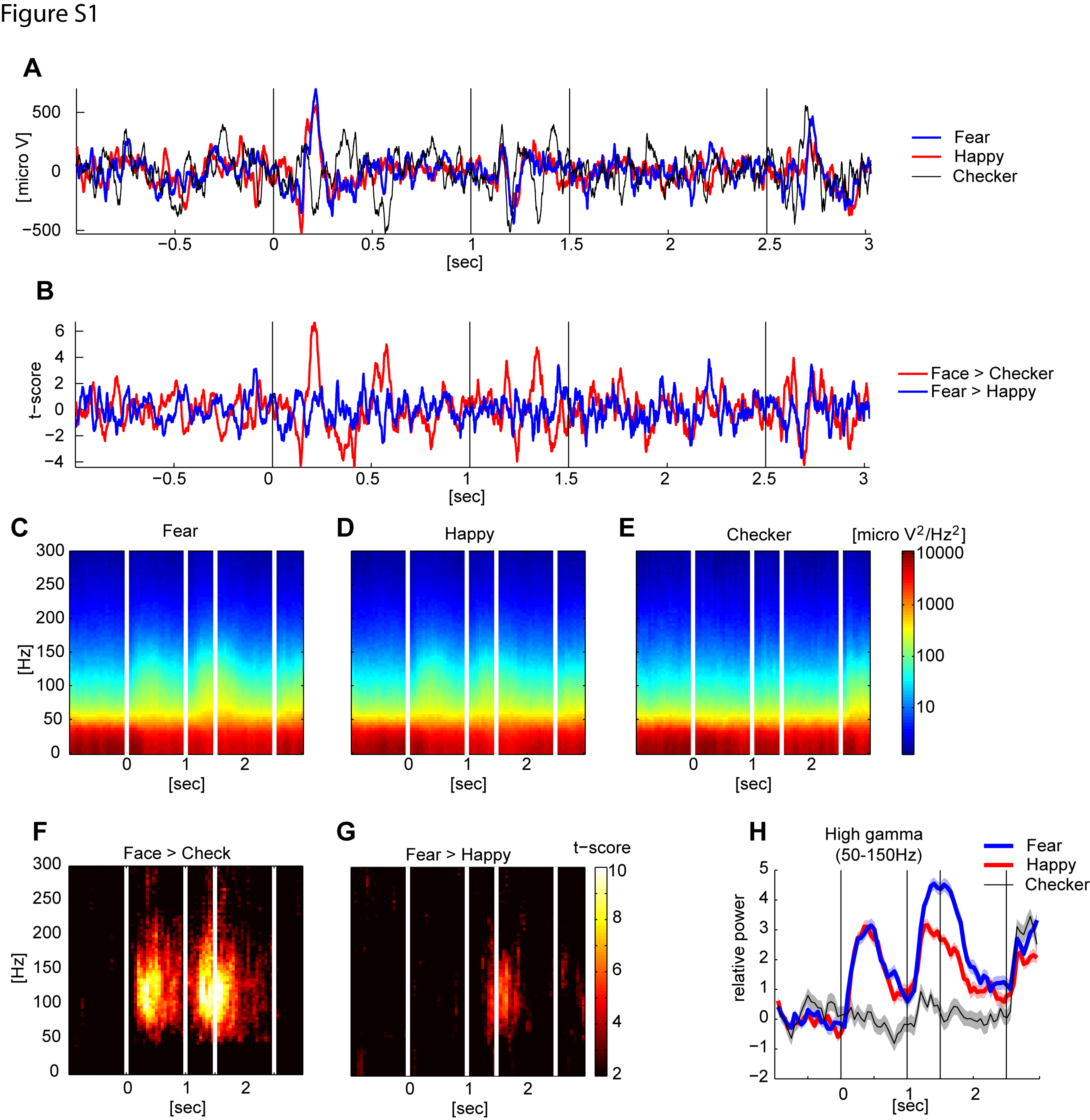

Supplement: Figure S1 — Standard ERP and spectrogram analysis (1) A–H) Analysis of channel 75 for subject 153 (session 1). A ERP analysis. The average traces of field potentials are shown for fearful faces (80 trials, thick blue), happy faces (80 trials, thick red) and checkerboards (40 trials, thin black). This electrode showed large positive potential to faces at around 200 msec from the onset of the stimuli (t = 0 sec), but no such clear peak around the onset of the dynamic morph (t = 1 sec). B T-score for the difference between face and checkerboard (red) and between fearful and happy faces (blue). C–E Mean time-frequency spectrogram for fearful (C), happy (D) and checkerboard (E) conditions. Increased power around 100 Hz is seen at just after the onset of both static (t = 0 sec) and morph (t = 1 sec) period for faces (C and D) but not for checkerboards (E). Mean of the spectrogram for each trial is color-coded in log-scale. See the bar at the right for color scale. F and G T-scores for the difference between faces and checkerboards (F) and between fearful and happy faces (G), showing strong difference between conditions in high frequencies, which was not evident in the ERP analysis (A and B). See the bar at the right for color scale. H Relative power increase in the high-gamma bands (50–150 Hz). Mean high gamma power for fearful (blue), happy (red) and checkerboard (black) conditions are plotted, with the shades indicating one standard error of the mean across trials. The high-gamma power for this electrode increased relative to the baseline to faces, but not to checkerboard, at the onset of the static period. During the morph period, it increased even higher for fearful than happy faces. (3.21 MB TIF) [file pone.0003892.s001.tif]

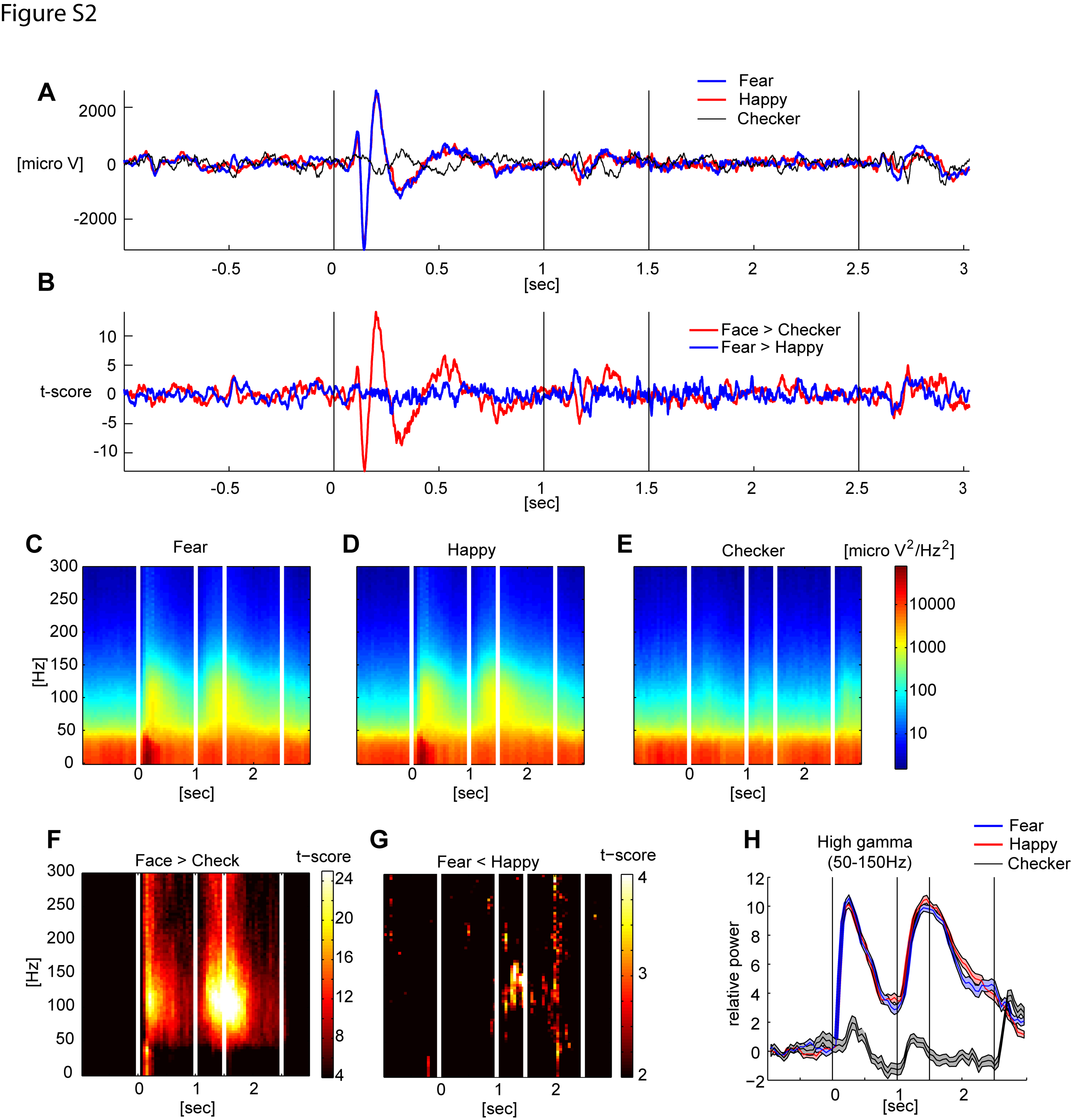

Supplement: Figure S2 — Standard ERP and spectrogram analysis (2) A–H) Analysis of channel 74 for subject 153 (session 1). A ERP analysis. This electrode showed much larger negative and positive potential to faces at around 200 msec from the onset of the stimuli than channel 75. B T-score for the difference between face and checkerboard (red) and between fearful and happy faces (blue). C–E Mean time-frequency spectrogram for fearful (C), happy (D) and checkerboard (E) conditions. (E). Mean of the spectrogram. (F and G) T-scores for the difference between faces and checkerboards (F) and between fearful and happy faces (G). See the bar at the right for color scale. H Relative power increase in the high-gamma bands (50–150 Hz). Mean high gamma power for fearful (blue), happy (red) and checkerboard (black) conditions are plotted, with the shades indicating one standard error of the mean across trials. (3.43 MB TIF) [file pone.0003892.s002.tif]

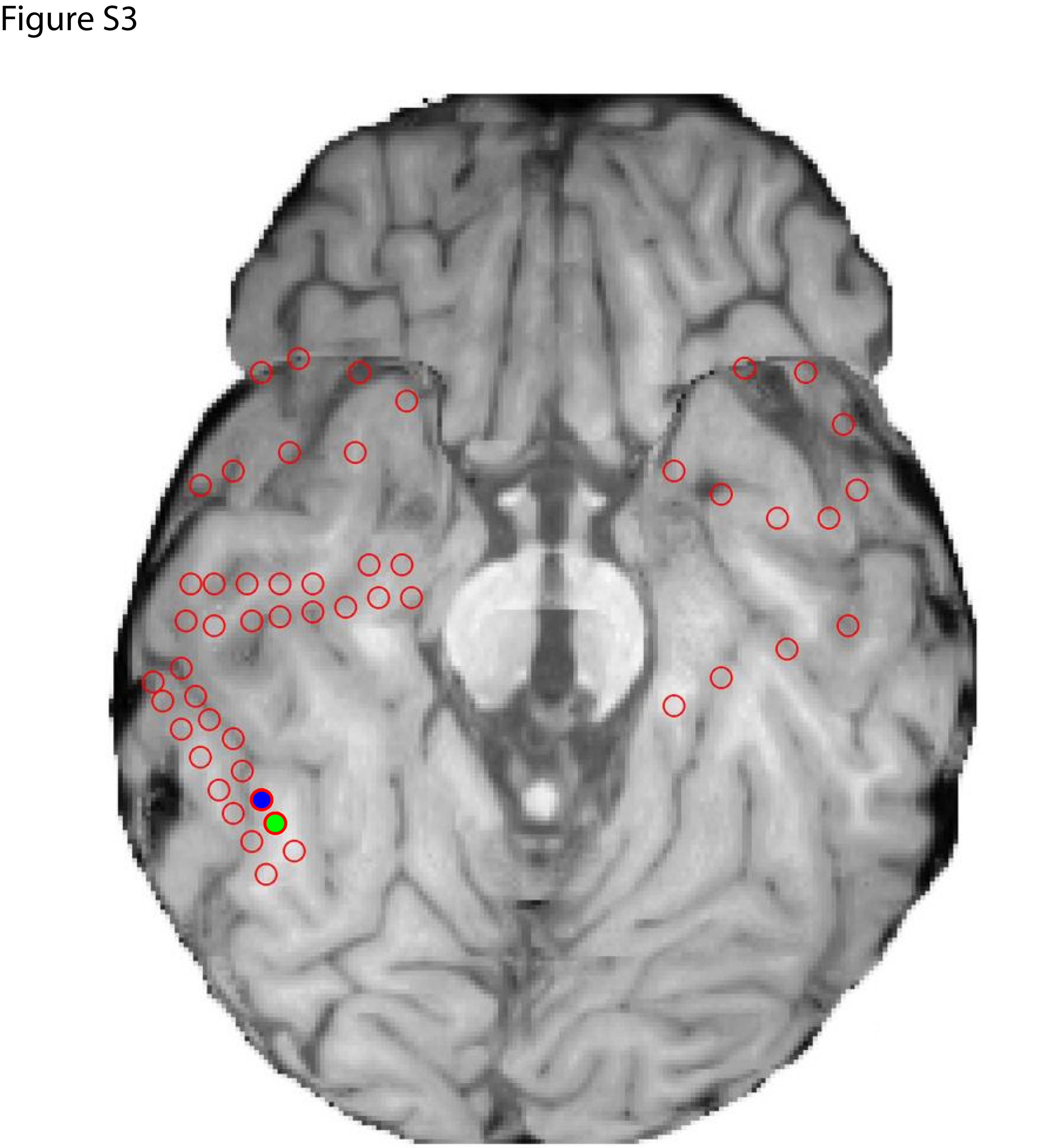

Supplement: Figure S3 — Electrode 74 and 75 are marked by green and blue circles, respectively, in the right ventral temporal cortex. (8.22 MB TIF) [file pone.0003892.s003.tif]
